# Supplementary material for: CRISPR/Cas9 Directed Reprogramming of iPSC for Accelerated Motor Neuron Differentiation Leads to Dysregulation of Neuronal Fate Patterning and Function
Source: Int J Mol Sci. 2023 Nov 10;24(22):16161. doi: 10.3390/ijms242216161 (PMC10671572; doi:10.3390/ijms242216161)
Supplement: Supplementary file 1 [file ijms-24-16161-s001.zip › ijms-2628965-supplementary.pdf]

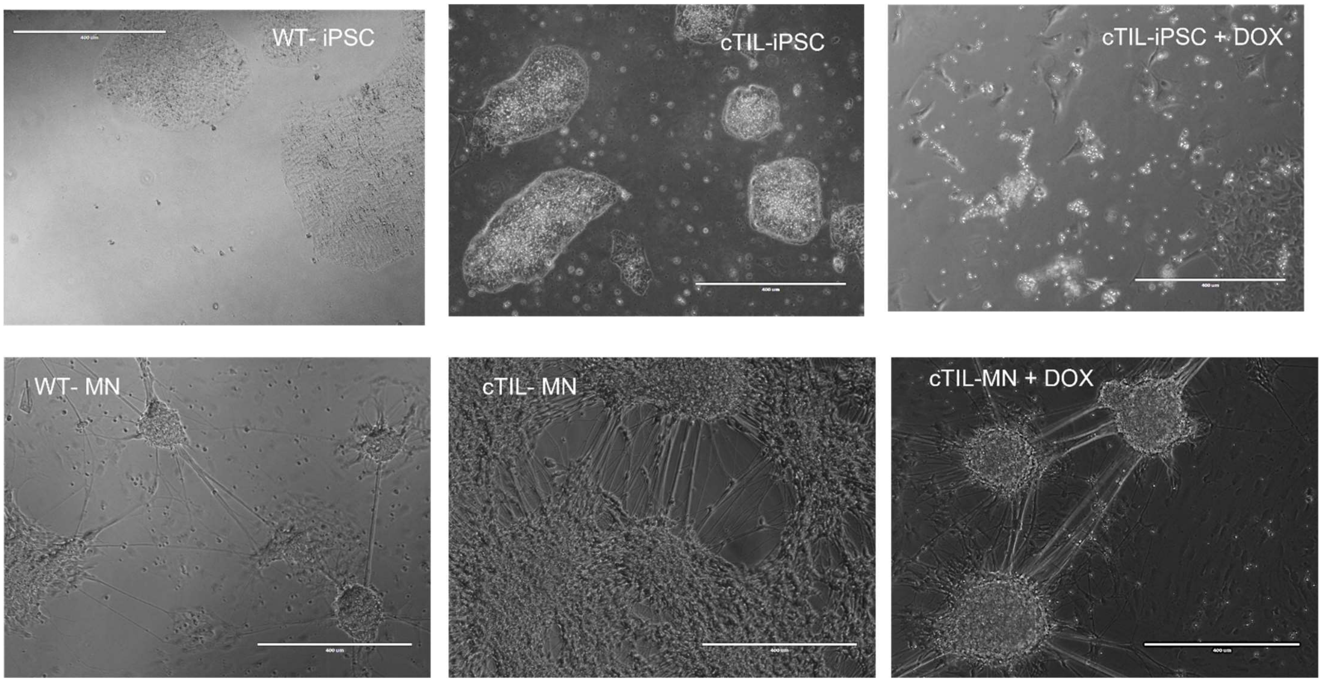

Supplementary Figure S1. Stimulation of transgene ISL1 and LHX3 with TET-ON system does not lead to unidirectional reprograming of iPSC to mature MNs. Bright field micrographs depicting morphology of wild type (WTC11) and cTIL -modified iPSC and corre-sponding mature motor neurons (MNs) derived via standardized protocol with differentiation media supplemented with neuron patterning molecules. Upper panel compares the morphology of unmodified iPSC (wild type, WT) and cTIL-modified iPSC without Dox and with Dox added to neuronal differentiation media without small molecules acting as MN patterning factors. The lower panel shows morphology of mature MNs derived from unmodified iPSC and cTIL-modified iPSC in media supplemented with neu-ron patterning factors, as described in the Methods and Figure 1 A schematic model.

Supplementary Table S1

Gene	Description	LogFC	p-adjusted	Time
NTRK2	neurotrophic receptor tyrosine kinase 2	8.88227	4.5978E-46	D0-D7
NTRK1	neurotrophic receptor tyrosine kinase 1	4.1871677	9.0515E-06	D0-D7
NTRK2	neurotrophic receptor tyrosine kinase 2	1.8007824	0.00057139	D13-D18
ERBB4	erb-b2 receptor tyrosine kinase 4	6.9227036	2.3277E-23	D7-D13
ERBB4	erb-b2 receptor tyrosine kinase 4	-3.3669753	3.1959E-06	D18-D28
FLT1	fms related receptor tyrosine kinase 1	-5.7320652	4.2518E-19	D0-D7
FLNC	filamin C	2.9279217	5.7635E-19	D0-D7
FLNC	filamin C	-1.4229274	0.00011955	D7-D13
KDR	kinase insert domain receptor	-6.4478754	2.8953E-18	D0-D7
KDR	kinase insert domain receptor	5.3643479	3.2684E-14	D18-D28
ERBB3	erb-b2 receptor tyrosine kinase 3	-2.8062189	3.5223E-17	D0-D7
ERBB3	erb-b2 receptor tyrosine kinase 3	-2.7958355	5.5391E-09	D7-D13
RASGRP2	RAS guanyl releasing protein 2	-5.3519632	3.085E-15	D0-D7
FOS	Fos proto-oncogene; AP-1 transcription factor subu	-2.7147721	8.884E-08	D7-D13
FOS	Fos proto-oncogene; AP-1 transcription factor subu	3.6919292	8.793E-15	D18-D28
FGFR1	fibroblast growth factor receptor 1	-1.8535704	1.7613E-12	D18-D28
CACNA2D3	calcium voltage-gated channel auxiliary subunit al	5.136164	2.2223E-12	D7-D13
DUSP6	dual specificity phosphatase 6	-2.6856203	5.7463E-12	D7-D13
FGFR4	fibroblast growth factor receptor 4	-2.9726949	2.5063E-11	D7-D13
MRAS	muscle RAS oncogene homolog	1.5217684	1.155E-10	D18-D28
EGFR	epidermal growth factor receptor	1.739147	0.00022585	D0-D7
EGFR	epidermal growth factor receptor	1.6603308	0.00081668	D7-D13
EGFR	epidermal growth factor receptor	-2.8071776	3.6943E-10	D18-D28
KITLG	KIT ligand	4.4290402	5.4219E-10	D0-D7
PPM1B	protein phosphatase; Mg2+/Mn2+ dependent 1B	-1.2845459	9.5905E-10	D0-D7
EFNA5	ephrin A5	3.7281509	1.8725E-09	D0-D7
NFATC3	nuclear factor of activated T cells 3	-1.2425845	2.3611E-09	D18-D28
IL1RAP	interleukin 1 receptor accessory protein	2.7526523	3.5887E-09	D18-D28
TNFRSF1A	TNF receptor superfamily member 1A	-1.3191865	0.00195182	D0-D7
TNFRSF1A	TNF receptor superfamily member 1A	2.4071472	3.9514E-09	D18-D28
FGF9	fibroblast growth factor 9	3.6133501	6.7822E-09	D0-D7
TGFB2	transforming growth factor beta 2	3.3889104	9.3355E-09	D0-D7
TGFB2	transforming growth factor beta 2	3.0763147	8.2648E-09	D7-D13
PTPN5	protein tyrosine phosphatase non-receptor type 5	2.4445749	1.4913E-08	D18-D28
TGFBR2	transforming growth factor beta receptor 2	2.9012801	2.0721E-08	D18-D28
FGFR3	fibroblast growth factor receptor 3	2.7895976	8.0706E-05	D0-D7
FGFR3	fibroblast growth factor receptor 3	-3.8222905	2.385E-08	D18-D28
ARRB1	arrestin beta 1	1.6662265	3.7008E-08	D0-D7
ARRB1	arrestin beta 1	1.4987329	3.849E-06	D13-D18
ARRB1	arrestin beta 1	1.0516625	0.00042454	D18-D28
PDGFC	platelet derived growth factor C	2.9939718	5.6208E-08	D0-D7
CACNA1E	calcium voltage-gated channel subunit alpha1 E	2.644264	5.8761E-08	D18-D28
CACNG8	calcium voltage-gated channel auxiliary subunit ga	-2.6038966	0.00053895	D0-D7

CACNG5	calcium voltage-gated channel auxiliary subunit ga	-6.2275401	1.4652E-07	D0-D7
CACNG7	calcium voltage-gated channel auxiliary subunit ga	-2.3644877	2.9701E-07	D0-D7
CACNG4	calcium voltage-gated channel auxiliary subunit ga	1.5320602	0.00012389	D7-D13
CACNA1B	calcium voltage-gated channel subunit alpha1 B	2.1171802	0.00022933	D7-D13
CACNG2	calcium voltage-gated channel auxiliary subunit ga	2.9925225	0.00124106	D13-D18
CACNG2	calcium voltage-gated channel auxiliary subunit ga	3.0882774	1.0393E-06	D18-D28
CACNG7	calcium voltage-gated channel auxiliary subunit ga	1.7836079	0.00025406	D18-D28
DUSP5	dual specificity phosphatase 5	-3.9097612	7.6268E-07	D7-D13
MAPKAPK2	MAPK activated protein kinase 2	0.9517235	4.951E-05	D0-D7
MAPKAPK2	MAPK activated protein kinase 2	-1.1455774	7.9786E-07	D18-D28
FGFR2	fibroblast growth factor receptor 2	-3.1058238	8.0964E-07	D18-D28
MYC	MYC proto-oncogene; bHLH transcription factor	-2.8424857	8.2798E-07	D0-D7
PDGFA	platelet derived growth factor subunit A	-3.9444139	9.074E-07	D7-D13
PDGFA	platelet derived growth factor subunit A	3.1698565	1.3847E-05	D18-D28
MECOM	MDS1 and EVI1 complex locus	2.124274	3.3779E-05	D0-D7
MECOM	MDS1 and EVI1 complex locus	-2.3966538	2.1038E-06	D18-D28
CACNA2D1	calcium voltage-gated channel auxiliary subunit al	-3.0994629	2.7524E-06	D0-D7
CACNA2D1	calcium voltage-gated channel auxiliary subunit al	2.7476582	0.00011623	D7-D13
KIT	KIT proto-oncogene; receptor tyrosine kinase	1.8017807	0.00019001	D0-D7
KIT	KIT proto-oncogene; receptor tyrosine kinase	-1.8502092	0.00029342	D7-D13
KIT	KIT proto-oncogene; receptor tyrosine kinase	-2.5618877	3.4086E-06	D18-D28
MAP4K2	mitogen-activated protein kinase kinase kinase kin	-1.8874145	3.6131E-06	D0-D7
MAP3K12	mitogen-activated protein kinase kinase kinase 12	1.2090284	3.6168E-06	D18-D28
CSF1	colony stimulating factor 1	2.421093	3.7443E-06	D0-D7
FLNB	filamin B	1.4359353	3.7861E-06	D18-D28
NTRK1	neurotrophic receptor tyrosine kinase 1	4.1871677	9.0515E-06	D0-D7
CACNB3	calcium voltage-gated channel auxiliary subunit be	1.9231339	1.2224E-05	D18-D28
FAS	Fas cell surface death receptor	2.233471	1.7457E-05	D0-D7
FAS	Fas cell surface death receptor	-1.8610346	0.00092509	D7-D13
CDC25B	cell division cycle 25B	-1.5032409	3.1998E-05	D18-D28
TRAF2	TNF receptor associated factor 2	-1.0326828	8.6001E-05	D0-D7
GADD45B	growth arrest and DNA damage inducible beta	4.2942885	0.00010322	D18-D28
RASGRP4	RAS guanyl releasing protein 4	-3.4675482	0.00011295	D0-D7
GADD45A	growth arrest and DNA damage inducible alpha	2.2220442	0.00018256	D0-D7
LAMTOR3	late endosomal/lysosomal adaptor; MAPK and MT	1.4851472	0.00019762	D18-D28
MAP3K20	mitogen-activated protein kinase kinase kinase 20	-2.5772947	0.00021098	D18-D28
FGF19	fibroblast growth factor 19	-5.9599903	0.00023948	D0-D7
RPS6KA3	ribosomal protein S6 kinase A3	1.3696894	0.00025118	D0-D7
MAPK13	mitogen-activated protein kinase 13	-3.08838	0.0005015	D0-D7
HSPB1	heat shock protein family B (small) member 1	-1.9289181	0.00054449	D0-D7
HSPB1	heat shock protein family B (small) member 1	1.9272991	0.00065762	D18-D28
MAP3K4	mitogen-activated protein kinase kinase kinase 4	-0.8959035	0.00056469	D18-D28
CACNB1	calcium voltage-gated channel auxiliary subunit be	1.9168376	0.00057486	D18-D28
RASGRF1	Ras protein specific guanine nucleotide releasing fa	3.1356054	0.00061751	D0-D7

AKT3	AKT serine/threonine kinase 3	2.154223	0.00064543	D7-D13
EGF	epidermal growth factor	2.6741765	0.00065483	D0-D7
EGF	epidermal growth factor	-2.5177495	0.00176827	D18-D28
IGF1R	insulin like growth factor 1 receptor	-1.8022136	0.00083364	D18-D28
PAK2	p21 (RAC1) activated kinase 2	0.4974171	0.00092386	D0-D7
MAPK11	mitogen-activated protein kinase 11	2.7329401	0.00124662	D0-D7
CASP3	caspase 3	1.0963061	0.00130608	D18-D28
FGF17	fibroblast growth factor 17	3.6348486	0.00132701	D0-D7
FGF17	fibroblast growth factor 17	2.641559	0.0014852	D18-D28
MAP3K1	mitogen-activated protein kinase kinase kinase 1	0.7530898	0.00136435	D0-D7
FGF5	fibroblast growth factor 5	5.6937525	0.00146246	D18-D28
NFKB1	nuclear factor kappa B subunit 1	1.5368973	0.00154879	D0-D7
ERBB2	erb-b2 receptor tyrosine kinase 2	-1.9443282	0.00191139	D0-D7
MAPK8	mitogen-activated protein kinase 8	-2.0954658	0.00198647	D18-D28
RAC3	Rac family small GTPase 3	-1.4482906	0.10115004	D0-D7

Gene	Description	LogFC	p-adjusted	Time
NTRK2	neurotrophic receptor tyrosine kinase 2	8.88227	4.60E-46	D0-D7
NTRK2	neurotrophic receptor tyrosine kinase 2	1.8007824	0.000571386	D13-D18
ERBB4	erb-b2 receptor tyrosine kinase 4	6.9227036	2.33E-23	D7-D13
ERBB4	erb-b2 receptor tyrosine kinase 4	-3.3669753	3.20E-06	D18-D28
HTR7	5-hydroxytryptamine receptor 7	-6.177465	2.14E-20	D0-D7
HTR7	5-hydroxytryptamine receptor 7	4.0824658	2.27E-09	D18-D28
FLT1	fms related receptor tyrosine kinase 1	-5.7320652	4.25E-19	D0-D7
KDR	kinase insert domain receptor	-6.4478754	2.90E-18	D0-D7
KDR	kinase insert domain receptor	5.3643479	3.27E-14	D18-D28
P2RX3	purinergic receptor P2X 3	1.5632292	0.008671179	D0-D7
P2RX3	purinergic receptor P2X 3	3.5169144	4.32E-18	D7-D13
P2RX3	purinergic receptor P2X 3	1.4521127	0.002191076	D13-D18
ERBB3	erb-b2 receptor tyrosine kinase 3	-2.8062189	3.52E-17	D0-D7
ERBB3	erb-b2 receptor tyrosine kinase 3	-2.7958355	5.54E-09	D7-D13
ITPR3	inositol 1;4;5-trisphosphate receptor type 3	-3.4909883	8.18E-17	D0-D7
RET	ret proto-oncogene	-4.2834617	1.28E-15	D0-D7
RET	ret proto-oncogene	3.1217043	1.44E-12	D18-D28
GRM1	glutamate metabotropic receptor 1	2.8199093	3.08E-05	D18-D28
GNA14	G protein subunit alpha 14	-5.4603243	5.35E-14	D0-D7
CHRM3	cholinergic receptor muscarinic 3	4.4302275	6.15E-14	D18-D28
FGFR1	fibroblast growth factor receptor 1	-0.9110739	0.002274665	D7-D13
FGFR1	fibroblast growth factor receptor 1	-1.8535704	1.76E-12	D18-D28
ATP2A3	ATPase sarcoplasmic/endoplasmic reticulum	-3.7220136	2.43E-12	D0-D7
ATP2A3	ATPase sarcoplasmic/endoplasmic reticulum	1.8720379	0.004483557	D7-D13
ATP2A3	ATPase sarcoplasmic/endoplasmic reticulum	-5.0739362	8.46E-05	D18-D28
FGFR4	fibroblast growth factor receptor 4	-1.1916313	0.005075853	D0-D7
FGFR4	fibroblast growth factor receptor 4	-2.9726949	2.51E-11	D7-D13
CCKBR	cholecystokinin B receptor	-4.4340386	7.80E-11	D0-D7
PDE1A	phosphodiesterase 1A	2.9650025	0.00151213	D0-D7
PDE1A	phosphodiesterase 1A	3.2209116	4.21E-05	D7-D13
PDE1A	phosphodiesterase 1A	-4.8373824	2.47E-10	D18-D28
ITPR2	inositol 1;4;5-trisphosphate receptor type 2	-3.9569499	2.90E-10	D0-D7
PLCE1	phospholipase C epsilon 1	4.1360108	3.19E-10	D0-D7
EGFR	epidermal growth factor receptor	1.739147	0.000225852	D0-D7
EGFR	epidermal growth factor receptor	1.6603308	0.000816683	D7-D13
EGFR	epidermal growth factor receptor	-2.8071776	3.69E-10	D18-D28
SLC8A1	solute carrier family 8 member A1	3.0958936	3.37E-09	D7-D13
FGF9	fibroblast growth factor 9	3.6133501	6.78E-09	D0-D7
FGFR3	fibroblast growth factor receptor 3	2.7895976	8.07E-05	D0-D7
FGFR3	fibroblast growth factor receptor 3	-3.8222905	2.39E-08	D18-D28
GRIN1	glutamate ionotropic receptor NMDA type su	3.9431799	3.69E-08	D18-D28
PDGFC	platelet derived growth factor C	2.9939718	5.62E-08	D0-D7
PDGFC	platelet derived growth factor C	1.6311438	0.009088785	D7-D13

CACNA1E	calcium voltage-gated channel subunit alpha1	1.587637	0.007197192	D7-D13
CACNA1E	calcium voltage-gated channel subunit alpha1	2.644264	5.88E-08	D18-D28
PLCB3	phospholipase C beta 3	-2.4812495	1.46E-07	D0-D7
CAMK2B	calcium/calmodulin dependent protein kinase	2.5515258	2.84E-07	D18-D28
NOS1	nitric oxide synthase 1	-3.8325012	6.02E-07	D0-D7
FGFR2	fibroblast growth factor receptor 2	-3.1058238	8.10E-07	D18-D28
PDGFA	platelet derived growth factor subunit A	-3.9444139	9.07E-07	D7-D13
PDGFA	platelet derived growth factor subunit A	3.1698565	1.38E-05	D18-D28
ADCY2	adenylate cyclase 2	-3.3586601	1.03E-06	D0-D7
ADCY2	adenylate cyclase 2	2.2691251	0.004367872	D7-D13
SLC8A3	solute carrier family 8 member A3	3.3348294	1.72E-06	D0-D7
SLC8A3	solute carrier family 8 member A3	1.5291393	0.008875811	D18-D28
ATP2B3	ATPase plasma membrane Ca2+ transporting	4.6552579	2.07E-06	D18-D28
CAMK2A	calcium/calmodulin dependent protein kinase	3.6297257	1.74E-05	D0-D7
CAMK2D	calcium/calmodulin dependent protein kinase	2.4776227	3.05E-06	D0-D7
CAMK2A	calcium/calmodulin dependent protein kinase	3.6297257	1.74E-05	D0-D7
CAMK2G	calcium/calmodulin dependent protein kinase	-0.6998203	0.00186652	D0-D7
CAMK4	calcium/calmodulin dependent protein kinase	1.9630544	4.94E-05	D7-D13
RYR1	ryanodine receptor 1	-2.1931211	3.42E-05	D0-D7
RYR2	ryanodine receptor 2	3.4913884	3.75E-06	D18-D28
GRPR	gastrin releasing peptide receptor	-3.634838	4.32E-06	D0-D7
ATP2B2	ATPase plasma membrane Ca2+ transporting	2.4145517	7.54E-06	D18-D28
NTRK1	neurotrophic receptor tyrosine kinase 1	4.1871677	9.05E-06	D0-D7
NTRK1	neurotrophic receptor tyrosine kinase 1	-2.3649505	0.005309894	D18-D28
GRIN2D	glutamate ionotropic receptor NMDA type su	-2.5464634	1.02E-05	D0-D7
GRIN2D	glutamate ionotropic receptor NMDA type su	1.7472436	0.003637442	D18-D28
PLCG2	phospholipase C gamma 2	-1.9009496	1.80E-05	D0-D7
ADRA1D	adrenoceptor alpha 1D	4.1272704	5.79E-05	D18-D28
EDNRB	endothelin receptor type B	1.7982777	0.010710313	D7-D13
EDNRB	endothelin receptor type B	-2.3739425	0.00012481	D18-D28
HTR2A	5-hydroxytryptamine receptor 2A	2.0985519	0.000201697	D18-D28
PTGER3	prostaglandin E receptor 3	3.2949466	0.000217684	D7-D13
CACNA1B	calcium voltage-gated channel subunit alpha1	2.1171802	0.000229332	D7-D13
FGF19	fibroblast growth factor 19	-5.9599903	0.000239484	D0-D7
HRH2	histamine receptor H2	-2.3512069	0.007508993	D0-D7
HRH2	histamine receptor H2	-4.3881151	0.000251157	D18-D28
MYLK	myosin light chain kinase	-1.9932382	0.000252543	D18-D28
ADCY8	adenylate cyclase 8	3.2588981	0.000316714	D7-D13
VDAC1	voltage dependent anion channel 1	-1.216347	0.00033444	D0-D7
PLCB1	phospholipase C beta 1	2.5741385	0.00038703	D0-D7
ATP2B1	ATPase plasma membrane Ca2+ transporting	1.4428461	0.000478206	D0-D7
PLCB2	phospholipase C beta 2	-2.2965118	0.000650156	D0-D7
EGF	epidermal growth factor	2.6741765	0.000654829	D0-D7
EGF	epidermal growth factor	-2.5177495	0.001768266	D18-D28

PHKA1	phosphorylase kinase regulatory subunit alph	1.2778975	0.000885055	D0-D7
NTRK3	neurotrophic receptor tyrosine kinase 3	1.5190976	0.001213945	D0-D7
NTRK3	neurotrophic receptor tyrosine kinase 3	1.5901428	0.001051593	D7-D13
CXCR4	C-X-C motif chemokine receptor 4	2.9330094	0.001076283	D0-D7
SLC8A2	solute carrier family 8 member A2	-3.0461997	0.001086587	D0-D7
SLC8A2	solute carrier family 8 member A2	2.8143064	0.004029173	D18-D28
FGF17	fibroblast growth factor 17	3.6348486	0.001327014	D0-D7
FGF17	fibroblast growth factor 17	2.641559	0.001485202	D18-D28
FGF5	fibroblast growth factor 5	5.6937525	0.001462456	D18-D28
CAMK2G	calcium/calmodulin dependent protein kinase	-0.6998203	0.00186652	D0-D7
ERBB2	erb-b2 receptor tyrosine kinase 2	-1.9443282	0.001911392	D0-D7
PDGFRB	platelet derived growth factor receptor beta	1.9504428	0.002226192	D18-D28
PDE1B	phosphodiesterase 1B	2.0153474	0.00870672	D0-D7
PDE1B	phosphodiesterase 1B	-2.4652831	0.002450414	D7-D13
ADRA1B	adrenoceptor alpha 1B	5.2505205	0.002889806	D0-D7
CALM2	calmodulin 2	1.0380034	0.003157836	D18-D28
TACR3	tachykinin receptor 3	-1.7318712	0.003194541	D0-D7
ADRA1A	adrenoceptor alpha 1A	3.9027325	0.003248004	D18-D28
MST1	macrophage stimulating 1	1.4482651	0.00364194	D18-D28
RYR3	ryanodine receptor 3	2.0600879	0.003654632	D0-D7
VEGFC	vascular endothelial growth factor C	2.453796	0.003686182	D7-D13
HRC	histidine rich calcium binding protein	-3.2993125	0.003900004	D0-D7
TRDN	triadin	-3.3252433	0.004123503	D0-D7
CHRM2	cholinergic receptor muscarinic 2	5.066906	0.00415318	D0-D7
CALM1	calmodulin 1	1.200185	0.004333244	D18-D28
SLC25A31	solute carrier family 25 member 31	5.3265982	0.004396948	D0-D7
PDGFD	platelet derived growth factor D	1.8743636	0.004658379	D18-D28
HTR2C	5-hydroxytryptamine receptor 2C	-3.228322	0.004748108	D0-D7
CACNA1D	calcium voltage-gated channel subunit alpha1	1.9281574	0.00548549	D0-D7
FGF1	fibroblast growth factor 1	-2.6098033	0.005857654	D0-D7
ADCY1	adenylate cyclase 1	0.7168792	0.005911127	D18-D28
FGF10	fibroblast growth factor 10	-2.6561533	0.006743216	D18-D28
PHKG2	phosphorylase kinase catalytic subunit gamm	-1.0707616	0.006953261	D0-D7
TNNC1	troponin C1; slow skeletal and cardiac type	-4.1962737	0.007129734	D7-D13
SPHK2	sphingosine kinase 2	-1.4584291	0.007132653	D0-D7
VEGFB	vascular endothelial growth factor B	-1.7684136	0.007252917	D0-D7
STIM1	stromal interaction molecule 1	0.8309992	0.007798559	D0-D7
PTGFR	prostaglandin F receptor	3.8266955	0.007911383	D18-D28
PRKCA	protein kinase C alpha	1.7943984	0.008573289	D0-D7
DRD1	dopamine receptor D1	7.8034365	0.008891529	D18-D28
PRKACA	protein kinase cAMP-activated catalytic subu	2.8758522	0.00964806	D13-D18
SLC25A4	solute carrier family 25 member 4	1.3962874	0.00969237	D18-D28
MCOLN2	mucolipin TRP cation channel 2	-1.9269193	0.010021052	D0-D7
CYSLTR2	cysteinyl leukotriene receptor 2	4.5362914	0.010121454	D7-D13

Gene	Description	LogFC	p-adjusted	Time
CNR1	cannabinoid receptor 1	3.302180901	9.73E-16	D0-D7
CNR1	cannabinoid receptor 1	3.286858239	1.26E-25	D7-D13
GABRA5	gamma-aminobutyric acid type A receptor subunit	-4.33140428	2.00E-20	D0-D7
GABRA5	gamma-aminobutyric acid type A receptor subunit	1.964794108	0.000137175	D18-D28
HTR7	5-hydroxytryptamine receptor 7	-6.17746505	2.14E-20	D0-D7
HTR7	5-hydroxytryptamine receptor 7	4.082465824	2.27E-09	D18-D28
APLNR	apelin receptor	3.208827929	2.53E-11	D0-D7
APLNR	apelin receptor	-4.74777872	5.30E-20	D7-D13
P2RX3	purinergic receptor P2X 3	3.516914417	4.32E-18	D7-D13
P2RX3	purinergic receptor P2X 3	1.452112729	0.002191076	D13-D18
GRID2	glutamate ionotropic receptor delta type subunit 2	-5.00680967	1.09E-15	D0-D7
GRID2	glutamate ionotropic receptor delta type subunit 2	2.638872665	9.95E-05	D18-D28
DRD2	dopamine receptor D2	6.358150766	1.48E-15	D0-D7
DRD2	dopamine receptor D2	-2.56864449	0.000132274	D18-D28
NPY1R	neuropeptide Y receptor Y1	-3.67602331	2.52E-15	D0-D7
NPY1R	neuropeptide Y receptor Y1	2.002608281	0.000147642	D18-D28
CHRNB4	cholinergic receptor nicotinic beta 4 subunit	2.909827054	9.15E-15	D0-D7
CHRNB4	cholinergic receptor nicotinic beta 4 subunit	-2.0305826	2.18E-07	D18-D28
NR3C1	nuclear receptor subfamily 3 group C member 1	-2.13666053	0.000109938	D0-D7
NR3C1	nuclear receptor subfamily 3 group C member 1	4.001445828	1.55E-14	D7-D13
NR3C1	nuclear receptor subfamily 3 group C member 1	-2.33199054	1.88E-05	D18-D28
GABRB3	gamma-aminobutyric acid type A receptor subunit	-3.21222464	1.97E-14	D0-D7
GABRB3	gamma-aminobutyric acid type A receptor subunit	2.081524155	7.03E-06	D7-D13
GABRB1	gamma-aminobutyric acid type A receptor subunit	5.509424436	5.45E-14	D7-D13
GABRB1	gamma-aminobutyric acid type A receptor subunit	-2.94589554	0.000859739	D13-D18
CHRM3	cholinergic receptor muscarinic 3	4.430227476	6.15E-14	D18-D28
CHRNA3	cholinergic receptor nicotinic alpha 3 subunit	2.498934037	1.12E-13	D0-D7
CHRNA3	cholinergic receptor nicotinic alpha 3 subunit	-1.39435502	5.00E-05	D18-D28
SSTR1	somatostatin receptor 1	8.347220454	4.41E-13	D18-D28
GRIA1	glutamate ionotropic receptor AMPA type subunit	3.773801329	5.47E-13	D0-D7
GRM4	glutamate metabotropic receptor 4	-4.29862559	8.37E-13	D0-D7
NTS	neurotensin	-9.52122218	7.01E-12	D0-D7
CCKBR	cholecystokinin B receptor	-4.4340386	7.80E-11	D0-D7
NPY5R	neuropeptide Y receptor Y5	-3.76631039	4.78E-10	D0-D7
NPY5R	neuropeptide Y receptor Y5	3.204181836	5.08E-05	D18-D28
HTR1E	5-hydroxytryptamine receptor 1E	5.765083081	5.84E-10	D18-D28
GLRA3	glycine receptor alpha 3	5.29254682	1.66E-09	D18-D28
LPAR3	lysophosphatidic acid receptor 3	-1.64370677	0.000764733	D0-D7
LPAR3	lysophosphatidic acid receptor 3	-3.61026401	1.81E-09	D7-D13
P2RY1	purinergic receptor P2Y1	-2.37226962	9.25E-09	D0-D7
GRIN2B	glutamate ionotropic receptor NMDA type subuni	4.034542268	2.56E-08	D18-D28
GRIN1	glutamate ionotropic receptor NMDA type subuni	3.943179939	3.69E-08	D18-D28
GRIA2	glutamate ionotropic receptor AMPA type subunit	3.802233924	7.76E-08	D7-D13

GRIA2	glutamate ionotropic receptor AMPA type subunit	2.040217859	0.000713097	D13-D18
GRIA2	glutamate ionotropic receptor AMPA type subunit	2.102485586	1.82E-05	D18-D28
HTR1D	5-hydroxytryptamine receptor 1D	3.880875361	1.03E-07	D0-D7
HTR1D	5-hydroxytryptamine receptor 1D	-3.82719626	6.93E-07	D18-D28
TRH	thyrotropin releasing hormone	5.055501219	2.57E-07	D18-D28
CHRNA5	cholinergic receptor nicotinic alpha 5 subunit	-2.16913037	2.73E-07	D18-D28
GLRB	glycine receptor beta	3.498364714	3.99E-07	D0-D7
GRIA3	glutamate ionotropic receptor AMPA type subunit	2.954700849	4.26E-07	D7-D13
ADCYAP1	adenylate cyclase activating polypeptide 1	3.705191323	7.62E-07	D13-D18
HCRTR2	hypocretin receptor 2	3.485746081	1.32E-06	D18-D28
S1PR1	sphingosine-1-phosphate receptor 1	3.838225674	1.53E-06	D0-D7
GRPR	gastrin releasing peptide receptor	-3.63483803	4.32E-06	D0-D7
GRIN2D	glutamate ionotropic receptor NMDA type subuni	-2.54646343	1.02E-05	D0-D7
GRIN3A	glutamate ionotropic receptor NMDA type subuni	2.274265537	1.13E-05	D18-D28
PTGER4	prostaglandin E receptor 4	3.10376649	1.28E-05	D18-D28
F2	coagulation factor II; thrombin	4.149282931	1.31E-05	D0-D7
NMU	neuromedin U	-3.29629585	2.05E-05	D18-D28
GRIA4	glutamate ionotropic receptor AMPA type subunit	-2.22368812	2.85E-05	D0-D7
GRIA4	glutamate ionotropic receptor AMPA type subunit	2.198761738	9.37E-05	D7-D13
GRM1	glutamate metabotropic receptor 1	2.819909301	3.08E-05	D18-D28
ADRA1D	adrenoceptor alpha 1D	4.127270423	5.79E-05	D18-D28
GLRA1	glycine receptor alpha 1	2.967428475	6.02E-05	D0-D7
GABRG1	gamma-aminobutyric acid type A receptor subunit	3.96010845	8.51E-05	D18-D28
GABRG2	gamma-aminobutyric acid type A receptor subunit	2.328574727	9.52E-05	D13-D18
GABRG2	gamma-aminobutyric acid type A receptor subunit	1.823038178	8.51E-05	D18-D28
GABRR2	gamma-aminobutyric acid type A receptor subunit	4.062352193	8.59E-05	D0-D7
MCHR1	melanin concentrating hormone receptor 1	-4.39419696	8.79E-05	D0-D7
GRIK2	glutamate ionotropic receptor kainate type subuni	2.734867375	0.000107548	D7-D13
ADRA2B	adrenoceptor alpha 2B	-2.73077381	0.000111589	D0-D7
EDNRB	endothelin receptor type B	-2.37394249	0.00012481	D18-D28
GPR156	G protein-coupled receptor 156	-2.75855523	0.000125227	D18-D28
GABRA3	gamma-aminobutyric acid type A receptor subunit	1.971521432	0.000174813	D18-D28
GRIK4	glutamate ionotropic receptor kainate type subuni	-2.54987838	0.000198627	D0-D7
HTR2A	5-hydroxytryptamine receptor 2A	2.098551877	0.000201697	D18-D28
GABRA2	gamma-aminobutyric acid type A receptor subunit	2.741023754	0.000202806	D18-D28
GABRB2	gamma-aminobutyric acid type A receptor subunit	2.448843168	0.000205827	D0-D7
PTGER3	prostaglandin E receptor 3	3.294946586	0.000217684	D7-D13
GHR	growth hormone receptor	2.512218713	0.000228086	D0-D7
TAC1	tachykinin precursor 1	3.931834981	0.000236671	D18-D28
HRH2	histamine receptor H2	-4.38811506	0.000251157	D18-D28
CHRNA1	cholinergic receptor nicotinic alpha 1 subunit	3.220303021	0.000315203	D18-D28
GRIK5	glutamate ionotropic receptor kainate type subuni	-1.50789117	0.000585871	D0-D7
GRIK5	glutamate ionotropic receptor kainate type subuni	-1.5290761	0.002142616	D7-D13
VIPR1	vasoactive intestinal peptide receptor 1	-2.55659158	0.000759443	D0-D7

F2RL1	F2R like trypsin receptor 1	-2.58197939	0.000770744	D7-D13
GRIK1	glutamate ionotropic receptor kainate type subuni	2.160869812	0.000796106	D0-D7
PARD3	par-3 family cell polarity regulator	-2.06486635	0.001381965	D18-D28
GRID1	glutamate ionotropic receptor delta type subunit 1	2.736745457	0.001466048	D0-D7
ADM	adrenomedullin	-2.77582402	0.001625868	D0-D7
ADM	adrenomedullin	3.606044725	0.002325252	D18-D28
GRIK3	glutamate ionotropic receptor kainate type subuni	-2.12712503	0.001629953	D18-D28
CRHR2	corticotropin releasing hormone receptor 2	-2.44946778	0.001680209	D18-D28
OPRK1	opioid receptor kappa 1	-3.60859084	0.001721168	D18-D28
ADRA2A	adrenoceptor alpha 2A	5.526810176	0.00209535	D0-D7
GIPR	gastric inhibitory polypeptide receptor	3.328095301	0.002173243	D18-D28

Gene	Description	LogFC	p-adjusted	Time
PLXNA2	plexin A2	7.4555832	1.06E-40	D0-D7
PLXNA2	plexin A2	-2.031211	0.000452287	D18-D28
EPHA3	EPH receptor A3	6.8439388	3.14E-37	D7-D13
DPYSL5	dihydropyrimidinase like 5	3.3730569	1.51E-35	D0-D7
EFNB2	ephrin B2	4.0577767	2.37E-32	D0-D7
PTCH1	patched 1	4.2484847	1.32E-31	D0-D7
PTCH1	patched 1	-1.756543	2.52E-06	D18-D28
SEMA3C	semaphorin 3C	6.3279462	2.14E-25	D0-D7
ABLIM3	actin binding LIM protein family member 3	3.666142	2.06E-24	D18-D28
RGMA	repulsive guidance molecule BMP co-receptor a	3.1699685	3.81E-23	D0-D7
RGMA	repulsive guidance molecule BMP co-receptor a	-1.414823	5.49E-05	D18-D28
BMP7	bone morphogenetic protein 7	3.7338339	5.36E-21	D0-D7
BOC	BOC cell adhesion associated; oncogene regula	3.6519528	6.61E-19	D0-D7
BOC	BOC cell adhesion associated; oncogene regula	-2.03256	2.93E-06	D18-D28
WNT4	Wnt family member 4	4.4163769	1.07E-18	D18-D28
NRP1	neuropilin 1	4.8760412	7.17E-18	D0-D7
EPHA5	EPH receptor A5	1.8579666	5.15E-05	D13-D18
EPHA5	EPH receptor A5	3.1553534	1.02E-17	D18-D28
SLIT1	slit guidance ligand 1	4.0577164	2.00E-16	D0-D7
SLIT1	slit guidance ligand 1	2.4589212	1.17E-07	D7-D13
UNC5B	unc-5 netrin receptor B	-2.726278	1.84E-15	D0-D7
UNC5B	unc-5 netrin receptor B	-2.349452	7.11E-09	D7-D13
UNC5B	unc-5 netrin receptor B	1.5039133	0.000414	D18-D28
EPHB3	EPH receptor B3	3.5978021	1.69E-14	D0-D7
WNT5A	Wnt family member 5A	3.1875875	5.11E-10	D0-D7
WNT5A	Wnt family member 5A	3.6304262	8.45E-14	D7-D13
DCC	DCC netrin 1 receptor	2.2437511	0.002194971	D0-D7
DCC	DCC netrin 1 receptor	4.8911275	1.46E-13	D7-D13
SRGAP3	SLIT-ROBO Rho GTPase activating protein 3	2.9931034	4.99E-13	D0-D7
SRGAP3	SLIT-ROBO Rho GTPase activating protein 3	1.5512343	0.001152635	D7-D13
EPHA7	EPH receptor A7	2.2808091	1.20E-11	D0-D7
FZD3	frizzled class receptor 3	1.3852612	1.21E-11	D0-D7
FZD3	frizzled class receptor 3	-0.893768	3.75E-05	D18-D28
PLXNC1	plexin C1	3.4560509	1.77E-11	D0-D7
SEMA3F	semaphorin 3F	-4.361813	2.73E-11	D0-D7
SEMA5B	semaphorin 5B	-2.173337	7.98E-11	D18-D28
SEMA6A	semaphorin 6A	-2.608852	2.15E-10	D0-D7
SEMA6A	semaphorin 6A	-1.458897	0.001264504	D18-D28
SLIT3	slit guidance ligand 3	3.1479361	2.35E-10	D7-D13
SLIT3	slit guidance ligand 3	2.139772	9.80E-05	D13-D18
SEMA3E	semaphorin 3E	-3.322236	3.20E-10	D7-D13
EFNA5	ephrin A5	3.7281509	1.87E-09	D0-D7
NFATC3	nuclear factor of activated T cells 3	-1.242584	2.36E-09	D18-D28

WNT5B	Wnt family member 5B	3.6943043	4.18E-08	D7-D13
WNT5B	Wnt family member 5B	-2.239568	0.001410133	D18-D28
CXCL12	C-X-C motif chemokine ligand 12	-4.804959	1.09E-07	D0-D7
CXCL12	C-X-C motif chemokine ligand 12	4.6483449	5.53E-07	D18-D28
SEMA4F	ssemaphorin 4F	1.5413344	1.11E-07	D18-D28
CAMK2B	calcium/calmodulin dependent protein kinase I	2.5515258	2.84E-07	D18-D28
ITGB1	integrin subunit beta 1	-1.180398	1.54E-06	D0-D7
ITGB1	integrin subunit beta 1	0.8945123	0.000513349	D18-D28
LRRC4C	leucine rich repeat containing 4C	3.0481671	2.87E-06	D0-D7
CAMK2D	calcium/calmodulin dependent protein kinase I	2.4776227	3.05E-06	D0-D7
UNC5C	unc-5 netrin receptor C	3.4579258	4.59E-06	D7-D13
PLXNA3	plexin A3	1.4704294	4.68E-06	D0-D7
SSH3	slingshot protein phosphatase 3	-2.368169	5.27E-06	D0-D7
EPHA8	EPH receptor A8	-3.724491	6.57E-06	D0-D7
EPHA8	EPH receptor A8	4.1445589	0.001382195	D18-D28
PIK3CD	phosphatidylinositol-4;5-bisphosphate 3-kinase	-1.981173	1.22E-05	D0-D7
PIK3CD	phosphatidylinositol-4;5-bisphosphate 3-kinase	2.0421683	1.11E-05	D18-D28
SEMA6D	semaphorin 6D	2.935058	1.12E-05	D7-D13
CAMK2A	calcium/calmodulin dependent protein kinase I	3.6297257	1.74E-05	D0-D7
PLCG2	phospholipase C gamma 2	-1.90095	1.80E-05	D0-D7
PIK3R1	phosphoinositide-3-kinase regulatory subunit 1	1.9165959	1.84E-05	D0-D7
ABLIM2	actin binding LIM protein family member 2	2.1998176	1.86E-05	D7-D13
FYN	FYN proto-oncogene; Src family tyrosine kinas	1.7084434	1.96E-05	D0-D7
NTNG1	netrin G1	3.4838763	2.07E-05	D13-D18
NTN1	netrin 1	2.4645416	3.41E-05	D0-D7
NTN1	netrin 1	2.2517479	0.000215092	D7-D13
RHOD	ras homolog family member D	-4.7653	5.88E-05	D0-D7
PTPN11	protein tyrosine phosphatase non-receptor type	-0.931659	0.000144629	D0-D7
ABLIM1	actin binding LIM protein 1	1.7143905	0.000181365	D18-D28
L1CAM	L1 cell adhesion molecule	1.9835491	0.000295421	D18-D28
PAK3	p21 (RAC1) activated kinase 3	1.8007917	0.000319969	D7-D13
MYL12B	myosin light chain 12B	1.4220522	0.000450582	D18-D28
PLXNA4	plexin A4	2.5016083	0.000492681	D13-D18
ROBO2	roundabout guidance receptor 2	-1.935829	0.001094884	D0-D7
ROBO2	roundabout guidance receptor 2	2.1429616	0.000571177	D7-D13
ROBO1	roundabout guidance receptor 1	1.7501016	0.000623942	D7-D13
DPYSL2	dihydropyrimidinase like 2	1.1263625	0.000737866	D18-D28
BMPR1B	bone morphogenetic protein receptor type 1B	2.9637636	0.000871988	D0-D7
PAK2	p21 (RAC1) activated kinase 2	0.4974171	0.000923861	D0-D7
SEMA3D	semaphorin 3D	-1.463013	0.000963853	D18-D28
BMPR2	bone morphogenetic protein receptor type 2	1.2056288	0.001013353	D7-D13
PAK5	p21 (RAC1) activated kinase 5	1.8513363	0.001761489	D0-D7
PAK5	p21 (RAC1) activated kinase 5	1.8751321	0.00102225	D7-D13
MYL12A	myosin light chain 12A	-1.595308	0.001030513	D0-D7

CXCR4	C-X-C motif chemokine receptor 4	2.9330094	0.001076283	D0-D7
PTK2	protein tyrosine kinase 2	-1.755081	0.001080391	D18-D28
PARD3	par-3 family cell polarity regulator	-2.064866	0.001381965	D18-D28
NTN4	netrin 4	1.671894	0.001548795	D7-D13
LIMK2	LIM domain kinase 2	0.79945	0.001623585	D18-D28
EPHB4	EPH receptor B4	-1.257105	0.001841581	D0-D7
CAMK2G	calcium/calmodulin dependent protein kinase I	-0.69982	0.00186652	D0-D7
SEMA4A	semaphorin 4A	-1.902545	0.001957459	D0-D7
RYK	receptor like tyrosine kinase	-0.746973	0.002093434	D18-D28

Gene	Description	LogFC	p-adjusted	Time
SLC5A7	solute carrier family 5 member 7	7.560729164	9.27E-08	D7-D13
SLC5A7	solute carrier family 5 member 7	3.510584028	2.95E-17	D13-D18
SLC5A7	solute carrier family 5 member 7	2.32643969	1.16E-09	D18-D28
ITPR3	inositol 1;4;5-trisphosphate receptor type 3	-3.49098828	8.18E-17	D0-D7
KCNJ12	potassium inwardly rectifying channel subfamily J r	3.708715056	1.34E-15	D0-D7
KCNJ12	potassium inwardly rectifying channel subfamily J r	-1.61151176	0.000291907	D18-D28
FOS	Fos proto-oncogene; AP-1 transcription factor subur	-2.71477208	8.88E-08	D7-D13
FOS	Fos proto-oncogene; AP-1 transcription factor subur	3.6919292	8.79E-15	D18-D28
CHRNB4	cholinergic receptor nicotinic beta 4 subunit	2.909827054	9.15E-15	D0-D7
CHRNB4	cholinergic receptor nicotinic beta 4 subunit	-2.0305826	2.18E-07	D18-D28
CHRM3	cholinergic receptor muscarinic 3	4.430227476	6.15E-14	D18-D28
CHRNA3	cholinergic receptor nicotinic alpha 3 subunit	2.498934037	1.12E-13	D0-D7
CHRNA3	cholinergic receptor nicotinic alpha 3 subunit	-1.39435502	5.00E-05	D18-D28
ITPR2	inositol 1;4;5-trisphosphate receptor type 2	-3.95694995	2.90E-10	D0-D7
PIK3R5	phosphoinositide-3-kinase regulatory subunit 5	-4.80855386	2.35E-08	D0-D7
CREB3L1	cAMP responsive element binding protein 3 like 1	-3.92509521	3.40E-08	D7-D13
CREB3L1	cAMP responsive element binding protein 3 like 1	3.554690755	7.72E-05	D18-D28
CHAT	choline O-acetyltransferase	3.721443261	6.37E-08	D13-D18
PLCB3	phospholipase C beta 3	-2.48124947	1.46E-07	D0-D7
CAMK2B	calcium/calmodulin dependent protein kinase II beta	2.551525784	2.84E-07	D18-D28
GNB5	G protein subunit beta 5	1.284669714	2.89E-07	D18-D28
ADCY2	adenylate cyclase 2	-3.35866007	1.03E-06	D0-D7
ADCY2	adenylate cyclase 2	2.269125132	0.004367872	D7-D13
CAMK2D	calcium/calmodulin dependent protein kinase II del	2.477622663	3.05E-06	D0-D7
ACHE	acetylcholinesterase (Cartwright blood group)	4.134748517	4.64E-06	D18-D28
PIK3CD	phosphatidylinositol-4;5-bisphosphate 3-kinase cata	-1.98117285	1.22E-05	D0-D7
PIK3CD	phosphatidylinositol-4;5-bisphosphate 3-kinase cata	2.042168335	1.11E-05	D18-D28
ADCY5	adenylate cyclase 5	-1.77098837	1.62E-05	D0-D7
CAMK2A	calcium/calmodulin dependent protein kinase II alp	3.629725689	1.74E-05	D0-D7
CREB5	cAMP responsive element binding protein 5	3.050694623	1.83E-05	D0-D7
PIK3R1	phosphoinositide-3-kinase regulatory subunit 1	1.916595917	1.84E-05	D0-D7
FYN	FYN proto-oncogene; Src family tyrosine kinase	1.708443397	1.96E-05	D0-D7
KCNQ3	potassium voltage-gated channel subfamily Q meml	-2.59979651	3.37E-05	D0-D7
KCNQ3	potassium voltage-gated channel subfamily Q meml	2.53574465	3.08E-05	D18-D28
KCNQ4	potassium voltage-gated channel subfamily Q meml	-2.02625827	4.33E-05	D18-D28
CAMK4	calcium/calmodulin dependent protein kinase IV	1.963054447	4.94E-05	D7-D13
GNB4	G protein subunit beta 4	-1.28635287	0.000127613	D0-D7
BCL2	BCL2 apoptosis regulator	2.081651837	0.000217577	D0-D7
GNG2	G protein subunit gamma 2	1.521420247	0.000223348	D18-D28
CACNA1B	calcium voltage-gated channel subunit alpha1 B	2.117180199	0.000229332	D7-D13
ADCY8	adenylate cyclase 8	3.258898062	0.000316714	D7-D13
PLCB1	phospholipase C beta 1	2.574138469	0.00038703	D0-D7
AKT3	AKT serine/threonine kinase 3	2.154222976	0.000645425	D7-D13

PLCB2	phospholipase C beta 2	-2.29651184	0.000650156	D0-D7
KCNQ2	potassium voltage-gated channel subfamily Q meml	2.035857889	0.004736327	D13-D18
KCNQ2	potassium voltage-gated channel subfamily Q meml	1.916267762	0.000728956	D18-D28
KCNJ3	potassium inwardly rectifying channel subfamily J r	-5.46245102	0.001258865	D0-D7
KCNJ3	potassium inwardly rectifying channel subfamily J r	5.191745884	0.005497039	D7-D13
CAMK2G	calcium/calmodulin dependent protein kinase II gar	-0.69982029	0.00186652	D0-D7
ADCY6	adenylate cyclase 6	1.191898035	0.003320181	D18-D28
CHRM2	cholinergic receptor muscarinic 2	5.066906028	0.00415318	D0-D7
CREB3L4	cAMP responsive element binding protein 3 like 4	-1.47750393	0.005392742	D0-D7
CACNA1D	calcium voltage-gated channel subunit alpha1 D	1.928157408	0.00548549	D0-D7
